# Nocardiosis: death from the ground

**DOI:** 10.25122/jml-2026-0076

**Published:** 2026-08

**Authors:** Amanda Shearer, Jonathan Balog, Jeffery Crick, Brittany Christopher, Renata Skov, Sahani Ambepitiya Waduge, George Pujalte

**Affiliations:** 1 Department of Family Medicine, Mayo Clinic, Jacksonville, Florida, USA; 2 Department of Orthopedic Surgery, Mayo Clinic, Jacksonville, Florida, USA

**Keywords:** brain, CNS, infection, lung, mass, nocardiosis

## Abstract

Nocardiosis is a rare but potentially life-threatening infection caused by aerobic, filamentous, gram-positive bacteria of the genus *Nocardia* found in soil and water. Although traditionally found in immunocompromised patients, up to one-third of cases occur in immunocompetent individuals, contributing to frequent diagnostic delays. Historically fatal before the antibiotic era, outcomes have substantially improved with modern antimicrobial therapy and diagnostic advances. However, nocardiosis remains underrecognized due to its nonspecific presentation and ability to mimic more common diseases. Pulmonary involvement is the most common manifestation, often resembling bacterial pneumonia or malignancy and presenting with cough, dyspnea, fever, weight loss, and/or lung masses. Hematogenous dissemination may occur, most commonly affecting the central nervous system, where brain abscesses can mimic neoplastic disease and may initially be asymptomatic. Cutaneous infections following direct inoculation may resemble refractory cellulitis or abscesses. Diagnosis requires a high index of suspicion, prolonged culture incubation, and species-level identification with susceptibility testing. We describe a 70-year-old man with pulmonary nocardiosis complicated by disseminated central nervous system involvement, initially suspected to represent malignancy, who achieved complete clinical and radiographic resolution with prolonged trimethoprim-sulfamethoxazole therapy. This case emphasizes the importance of early consideration of nocardiosis in patients with persistent pulmonary lesions and neurologic symptoms. We aimed to increase clinical awareness of nocardiosis, summarize its diverse presentations, and outline diagnostic and therapeutic principles to improve recognition, management, and outcomes in primary care and specialty settings. Early diagnosis and treatment are essential to reducing morbidity.

## INTRODUCTION

Nocardiosis is a rare infection seen in both immunocompromised and immunocompetent patients [1-3]. There are over 50 identified *Nocardia* species [2]. *Nocardia* are a group of bacteria found in soil, as well as fresh and salt water [2]. Before advances in antimicrobial testing and medical care, the mortality rate from nocardiosis was nearly 100% [1]. Thankfully, this rate has drastically improved over the years [4]. Understanding nocardiosis and including it in the differential diagnosis is vital for primary care physicians and various specialists (eg, pulmonologists, neurologists, infectious disease specialists) [2].

Nocardiosis, known as the “great masquerader,” is primarily recognized for causing pulmonary disease in immunocompromised patients, mimicking pneumonia and often requiring prolonged antibiotic treatment [5]. The disease typically progresses unless identified and treated [2]. Other common sites of infection are the central nervous system (CNS), which can mimic brain tumors, and the skin, which can mimic difficult-to-treat cellulitis or abscesses [1].

## CLINICAL VIGNETTE

A 70-year-old African American male nonsmoker with a history of diabetes and hypertension presented with a few weeks of cough and mild shortness of breath, without chest pain or fever. Chest radiography was performed for prolonged cough and dyspnea, revealing a mass in the lung fields near the bronchi, consistent with infection or possible obstruction. Augmentin was started, but the patient remained symptomatic. Computed tomography of his lungs revealed a persistent mass. Levaquin was initiated, and he was referred to the pulmonology department for bronchoscopy.

The pulmonologist evaluated the patient, continued the same antibiotic regimen, and performed a bronchoscopy with biopsy. Before the communication of biopsy results, the patient developed confusion, resulting in two automobile accidents within 2 days, a lower extremity venous thrombus, and became unresponsive. He was transferred to the emergency department, where multiple brain lesions were identified.

A neurologist and pulmonologist were consulted regarding the brain and lung masses. The pulmonologist reviewed the biopsy report, which identified *Nocardia* species (nocardiosis) without malignancy. The brain lesions were thought to be disseminated nocardiosis.

After consulting with the infectious disease department, trimethoprim-sulfamethoxazole was initiated. Over several weeks, the patient improved while hospitalized in the intensive care unit. He was discharged home with trimethoprim-sulfamethoxazole for 6 months and continued outpatient monitoring. The brain and lung masses resolved completely, and the patient remained asymptomatic after treatment.

## DISCUSSION

Nocardiosis is a difficult-to-diagnose and often underrecognized infection caused by aerobic, branching, filamentous actinomycetes of the genus *Nocardia*. These organisms are found in soil and water and typically cause infection through inhalation or direct tissue inoculation, particularly in individuals with impaired cell-mediated immunity. While solid organ transplant recipients, patients receiving corticosteroids, and those with HIV or malignancy are traditionally considered at highest risk, a substantial proportion of cases occur in immunocompetent individuals, reflecting a broader and more heterogeneous clinical spectrum than previously recognized [4,6].

Pulmonary involvement is the most common manifestation of nocardiosis. On imaging, pulmonary disease may resemble bacterial pneumonia, tuberculosis, or malignancy, often leading to diagnostic delay [7]. From the lungs, *Nocardia* may disseminate hematogenously, most commonly to the CNS, where cerebral abscesses can cause considerable morbidity and mortality [5]. Cutaneous and subcutaneous disease may also occur following direct inoculation, typically in immunocompetent patients, and can present as actinomycetoma or lymphocutaneous infection [4].

Emerging data suggest that the incidence of nocardiosis may be rising, likely due to increased use of immunosuppressive therapies, enhanced diagnostic capabilities, and greater clinical awareness [6]. Advances in molecular diagnostics, including 16S rRNA, *secA1*, and *rpoB* gene sequencing; matrix-assisted desorption/ionization-time-of-flight mass spectrometry; and genome-based identification, have improved diagnostic precision [4]. Species-level identification is critical because antimicrobial susceptibilities, virulence, and clinical outcomes vary substantially among *Nocardia* species [4].

First-line therapy typically consists of trimethoprimsulfamethoxazole, often combined with agents such as amikacin, imipenem, or third-generation cephalosporins in cases of severe or disseminated disease [7,8]. Treatment courses are prolonged, usually 6 to 12 months or longer for CNS involvement, to minimize relapse risk. Management is guided by antimicrobial susceptibility testing, clinical response, and toxicity monitoring [6,8]. Given the organism’s nonspecific presentation, slow culture growth, and expanding taxonomy, early recognition and prompt involvement of infectious disease specialists remain essential for improving outcomes [6].

Despite progress, uncertainties remain, including optimal treatment duration, prophylaxis strategies for high-risk populations, emerging resistance patterns, and the clinical role of next-generation sequencing. Continued integration of clinical, microbiologic, and genomic data is necessary to refine diagnosis, guide targeted therapy, and reduce morbidity and mortality associated with this underrecognized pathogen [5].

*Nocardia* species are environmental, filamentous, gram-positive organisms found in soil, water, and decaying vegetation [9,10]. Infection typically occurs via inhalation or through breaches in the skin. Although rare, with an estimated incidence of 0.3 to 0.8 cases per 100,000 individuals, nocardiosis can lead to severe disease, particularly in patients with impaired cellular immunity [9]. The highest risk groups include transplant recipients and those receiving corticosteroids or chemotherapy. At the same time, chronic lung diseases such as bronchiectasis and cystic fibrosis further increase susceptibility, especially in patients using inhaled corticosteroids. In previous studies, solid organ transplantation accounted for up to 40% of cases. Nevertheless, up to 20% of infections occur in seemingly immunocompetent individuals, some of whom are later found to have undiagnosed immune abnormalities such as chronic granulomatous disease or anti–granulocyte-macrophage colony-stimulating factor antibodies [9].

Large epidemiologic studies have demonstrated that nocardiosis predominantly affects older adults, with a mean age of 55 to 62 years [3,4,6]. Men account for approximately 60% to 70% of reported cases. Pulmonary involvement occurs in roughly 80% of patients, while skin and soft tissue infections account for 10% to 20%. Disseminated disease, particularly involving the CNS, is associated with higher mortality, with rates around 23% [3]. Notably, HIV infection has become a less common risk factor, likely due to effective antiretroviral therapy [3,9].

## CONCLUSION

Nocardiosis is an uncommon but clinically relevant infection that may present in the primary care setting. Although it primarily affects immunocompromised patients, both immunocompetent and immunocompromised individuals are susceptible. Pulmonary involvement is the most common manifestation, followed by CNS and skin infections. Management typically involves prolonged courses of antimicrobial therapy. Given its nonspecific presentation and potential for morbidity, ongoing clinical vigilance is essential for timely recognition and treatment of this underdiagnosed infection.

## Personal thanks

The Scientific Publications staff at Mayo Clinic provided copyediting, proofreading, administrative, and clerical support.
